# Readiness and Perception Toward Artificial Intelligence Among Undergraduate Medical Students at a Tertiary Care Teaching Institute: A Cross-Sectional Study

**DOI:** 10.7759/cureus.113804

**Published:** 2026-08-02

**Authors:** Kumarjiv K Shreshthi, Jaykumar H Nimavat, Milindkumar H Makwana, Pratik K Jasani, Kishor M Sochaliya, Meghadeepsinh I Chudasama

**Affiliations:** 1 Department of Community Medicine, Shrimad Rajchandra Sarvamangal Hospital and C U Shah Medical College, Surendranagar, IND

**Keywords:** ai readiness, artificial intelligence, cross-sectional study, india, mairs-ms, medical education, medical students, perception

## Abstract

Background

The growing integration of artificial intelligence (AI) into healthcare and medical education has created an urgent need to evaluate how prepared undergraduate students are to engage with these technologies. Medical graduates will increasingly encounter AI-driven tools across clinical and educational settings, yet systematic assessment of their readiness and perceptions remains limited, particularly in India. This study aimed to assess AI readiness and perceptions among undergraduate medical students and to examine how readiness varied in relation to sociodemographic characteristics, prior AI training, and patterns of AI tool utilization.

Methods

This cross-sectional study enrolled 310 undergraduate Bachelor of Medicine, Bachelor of Surgery (MBBS) students at a tertiary care teaching institution in Surendranagar, Gujarat, between September and November 2025. AI readiness and perception were assessed using the Medical Artificial Intelligence Readiness Scale for Medical Students (MAIRS-MS) and a separately developed, validated 10-item questionnaire, respectively. Descriptive statistics were summarized as means, standard deviations, frequencies, and percentages.

Participants were categorized as having poor (≤66), average (67-78), or good (≥79) readiness using cut-offs derived from the 33.33rd and 66.67th percentiles of the observed MAIRS-MS score distribution. The same percentile-based approach was applied to each MAIRS-MS domain score. Pearson's chi-square test or Fisher's exact test, as appropriate, was used to examine associations between categorical variables, while Spearman's rank correlation coefficient was used to assess relationships between readiness scores and selected variables. A two-sided p-value of less than 0.05 was considered statistically significant.

Results

The mean age of the study participants was 20.03 ± 1.65 years, and 182 (58.71%) were female subjects. Using the 33.33rd and 66.67th percentiles of the observed MAIRS-MS score distribution, 169 of 238 participants (71.0%) were classified as having average AI readiness. Previous exposure to AI training (χ² =6.33, p=0.042) was significantly associated with the ethics domain of AI readiness. Total AI readiness showed extremely weak positive correlations with age (r=0.087, p=0.181) and academic year of study (r=0.057, p=0.381), with neither relationship reaching statistical significance. Among all 310 participants, more than half of the students perceived AI as useful for several educational purposes, including teaching (n=165, 53.23%), assignment preparation (n=162, 52.26%), self-learning (n=166, 53.55%), understanding complex concepts (n=170, 54.84%), and clinical case scenarios (n=161, 51.93%). Substantial proportions also expressed concerns regarding misleading information (n=151, 48.70%), potential effects on clinical skills and critical thinking (n=151, 48.71%), and data privacy (n=133, 42.90%).

Conclusion

Overall, undergraduate medical students demonstrated an average level of AI readiness and generally mixed-to-positive perceptions toward artificial intelligence, with a considerable proportion of students remaining neutral across several items. Previous AI training or exposure was significantly associated with the ethics domain of AI readiness. An extremely weak positive correlation was observed between the total readiness score and both age and academic year of study.

## Introduction

Artificial intelligence (AI) has emerged as a transformative force across multiple domains of healthcare, supporting a broad range of clinical and public health activities, including diagnostic imaging, disease surveillance, clinical decision support, and individualized treatment planning [1-3]. Notably, deep-learning algorithms have demonstrated diagnostic performance comparable to that of experienced clinicians in specific domains, including dermatology and radiology [4,5]. A large international survey of radiologists and radiology residents reported widespread recognition of the potential of AI in diagnostic settings and increasing acceptance of AI-assisted workflows [6]. Rather than replacing clinical expertise, AI is increasingly used to support physicians in data interpretation, workload prioritization, and evidence-based decision-making [1,6,7].

These advances carry significant implications for how undergraduate medical education should evolve. As AI-enabled tools become embedded in hospital workflows and primary care practice, graduating physicians will need more than passive familiarity with these technologies. They must be able to evaluate the reliability of AI-generated outputs, identify the sources and consequences of algorithmic bias, safeguard patient data, and engage critically with the ethical dimensions of automated clinical recommendations [8-10]. This expectation has led several researchers and educators to call for the formal integration of AI literacy, understood as the synthesis of conceptual knowledge, applied skill, and ethical reasoning, into the core competencies of undergraduate medical curricula [8,11].

Karaca et al. addressed this measurement need by developing and validating the Medical Artificial Intelligence Readiness Scale for Medical Students (MAIRS-MS), a 22-item instrument designed to assess medical students' readiness for AI [12]. The scale comprises four subscales: cognition, ability, vision, and ethics. Together, these dimensions provide a structured assessment of different aspects of AI readiness and may help identify areas requiring further educational development. Survey-based evidence from multiple countries indicates that although medical students broadly view AI as a promising educational resource, their actual preparedness to apply it in clinical contexts is inconsistent. Findings from the United Kingdom, Malaysia, Indonesia, the UAE, and India converge on a common theme: students express recognition of AI's educational potential yet simultaneously report deficiencies in practical competence and ethical understanding [10,13-18]. A narrative systematic review and a scoping review both concluded that current undergraduate medical programs have incorporated AI-related content unevenly and urged the development of curricula that integrate technical foundations with applied ethics and clinical reasoning [19,20].

Within India, AI adoption in healthcare has accelerated substantially, with applications now deployed across diagnostic radiology, infectious disease monitoring, telemedicine platforms, and clinical decision assistance systems. This trajectory intensifies the responsibility of medical schools to produce graduates who can navigate these tools with both proficiency and discernment, recognizing their limitations and upholding patient-centered ethical standards. Although a few recent Indian studies have explored medical students' readiness and attitudes toward AI, much of the available evidence originates from metropolitan centres or major urban institutions, with limited evidence from other urban settings, particularly in Gujarat [10,17,21].

The present study was designed to address this regional evidence gap. Its objectives were: (1) to assess AI readiness and perceptions among undergraduate Bachelor of Medicine, Bachelor of Surgery (MBBS) students; (2) to examine the association of AI readiness with sociodemographic characteristics, prior AI training, and AI tool utilization - including both categorical associations (Pearson's chi-square) and rank correlations (Spearman's rho) with continuous variables.

## Materials and methods

Study design and setting

A cross-sectional survey was conducted among undergraduate medical students of Shrimad Rajchandra Sarvamangal Hospital & C U Shah Medical College, a tertiary care teaching institute in Surendranagar district, Gujarat, between September and November 2025. This study was conducted and reported in accordance with the Strengthening the Reporting of Observational Studies in Epidemiology (STROBE) guidelines for cross-sectional studies [22].

Sample size determination 

This institute enrolls 100 undergraduate students annually in its MBBS course. All undergraduate MBBS students from the first year (Phase I) to the final year (Phase III Part II) were invited to participate. The total target population consisted of 400 students, as the college enrolls 100 students each year, with a cumulative total of 400 students across all four phases. A universal (census) sampling approach was employed, whereby all eligible undergraduate MBBS students across all four academic years were invited to participate.

Inclusion and exclusion criteria

All undergraduate MBBS students (first MBBS, second MBBS, third MBBS part I, third MBBS part II) enrolled at the tertiary care teaching institute who were present during the data collection period and provided electronic informed consent through Google Forms (Google, California, US) were included in the study. Students who were absent during the data collection period or did not provide electronic informed consent were excluded. A total of 310 students met the eligibility criteria and were included in the final analysis. Of the 90 who were not included, 72 were absent on the day of data collection, and 18 did not provide electronic informed consent.

Ethical considerations

Before initiating the study, approval was obtained from the Institutional Ethics Committee (Human Research) of C U Shah Medical College and Shrimad Rajchandra Sarvamangal Hospital (CUSMC/IEC(HR)/Pro. Approval-RP-34/2025/OUT- 123/2025).

Data collection instruments

Data were collected using a self-administered Google Forms-based questionnaire. The survey form included four sections: sociodemographic information, variables related to exposure and utilization of AI tools, the MAIRS-MS, and items assessing perception toward AI. Sociodemographic characteristics recorded were age, gender, academic year of study, and place of residence. AI readiness was operationalized using the MAIRS-MS [12], a 22-item, psychometrically validated self-report scale with four subscales, cognition, ability, vision, and ethics, each employing a five-point Likert response format (1=strongly disagree to 5=strongly agree). The composite score ranges from 22 to 110, with higher values reflecting greater readiness for engagement with AI in medicine. Internal consistency of the original scale was reported as Cronbach's alpha =0.87 [12]. To assess perception toward AI, a separate pre-tested and validated questionnaire was used. The 10-item perception questionnaire was developed in consultation with senior faculty from the Department of Community Medicine and was pilot-tested among 30 MBBS students before the main data collection. Based on the feedback obtained, minor revisions were made to improve the clarity and wording of selected questions. The pilot study participants were excluded from the final analysis.

Responses to each item were scored on a five-point Likert scale: strongly disagree (1), disagree (2), neutral (3), agree (4), and strongly agree (5). The summed score ranged from 10 to 50, with higher scores indicating a more positive perception of AI. The questionnaire demonstrated strong internal consistency (Cronbach's alpha = 0.933), and its content validity was assessed by a panel of senior faculty members from the Department of Community Medicine, all holding postgraduate qualifications in Preventive and Social Medicine, with experience in epidemiological research and medical education. Higher scores on both instruments indicated greater AI readiness and a more positive perception toward AI, respectively. Since the MAIRS-MS was designed to assess readiness among individuals with prior AI experience, it was administered only to participants who reported having used AI tools for academic purposes in the preceding 12 months (n=238), whereas the perception questionnaire was administered to all 310 participants.

Data collection procedure

On a designated date, students received the Google Forms survey link. Before beginning the questionnaire, they were provided with information about the study purpose, voluntary participation, confidentiality, and electronic informed consent. Only students who provided consent could proceed with the questionnaire. Participants were informed that their responses would remain anonymous and that no individual identifiers would be linked to the collected data. The Google Form required a response to each item before submission; therefore, no incomplete questionnaires were included in the analysis.

Statistical analysis

Raw data were collated in Microsoft Excel 2024 (Microsoft Corporation, Redmond, WA, USA) and transferred to IBM SPSS Statistics for Windows, Version 25 (Released 2017; IBM Corp., Armonk, New York, United States) for all inferential analyses. Continuous variables are reported as mean ± standard deviation, whereas categorical variables are reported as frequencies and percentages. For the chi-square analysis, participants were classified as having poor, average, or good readiness using the 33.33rd and 66.67th percentiles of the observed MAIRS-MS score distribution as cut-off points. The 33.33rd and 66.67th percentile cut-offs were chosen because the original developers of the MAIRS-MS [12] did not establish fixed normative cut-off values for categorizing readiness levels. In the absence of externally validated thresholds, a tertile-based approach using the observed score distribution is methodologically appropriate. It distributes participants proportionally across categories, preserves statistical power for chi-square analyses, and avoids arbitrary fixed thresholds. This approach has also been adopted by Dhurandhar et al. in a comparable study using the same scale [17]. For the total MAIRS-MS score, participants were categorized as having poor (≤66), average (67-78), and good (≥79) readiness. The same percentile-based approach was applied separately to each MAIRS-MS domain score. Categorical associations were evaluated using Pearson's chi-square test; Fisher's exact test replaced chi-square when any expected cell count fell below five. The Kolmogorov-Smirnov test indicated that the outcome scores did not follow a normal distribution (p<0.001); therefore, non-parametric methods were used. Spearman's rank correlation coefficient (rho) was calculated to assess the relationship between readiness scores and continuous or ordinal variables. A two-tailed p-value <0.05 was considered statistically significant.

## Results

Of the 400 eligible students, 310 completed the survey (response rate: 77.5%). The mean age was 20.03 ± 1.65 years. Among respondents, 182 (58.71%) were female participants and 128 (41.29%) were male participants. Regarding place of residence, 240 (77.42%) belonged to urban areas and 70 (22.58%) to rural areas. The distribution across academic years was as follows: 100 (32.26%) from first MBBS, 60 (19.35%) from second MBBS, 102 (32.90%) from third MBBS Part I, and 48 (15.48%) from third MBBS Part II (Table 1).

**Table 1:** Demographic characteristics of participants (n=310) MBBS: Bachelor of Medicine and Bachelor of Surgery

Variable	Frequency	Percentage
Age	-	-
17–20	203	65.48%
21–24	107	34.52%
Gender	-	-
Female	182	58.71%
Male	128	41.29%
Place of Residence	-	-
Urban	240	77.42%
Rural	70	22.58%
Academic year of study	-	-
1^st^ MBBS	100	32.26%
2^nd^ MBBS	60	19.35%
3^rd^ MBBS part I	102	32.90%
3^rd^ MBBS part II	48	15.48%

Among the 310 participants, 238 (76.77%) had used AI for academic purposes during the past 12 months. The majority (n=227, 95.38%) used the free version of AI tools. ChatGPT (n=221, 92.86%) was the most commonly used AI tool, followed by Gemini (n=114, 47.90%) and Google Assistant (n=108, 45.38%). Among the 238 AI users, 86 (36.13%) used AI tools daily, 49 (20.59%) on alternate days, 19 (7.98%) weekly, and 84 (35.29%) occasionally. Only 63 (26.47%) participants had received any formal training in AI. Social media (n=192, 80.67%) was the most common source of information about AI, followed by friends (n=131, 55.04%). The most frequently reported challenge was the need for paid versions (n=159, 66.81%), followed by faulty data (n=72, 30.25%) and poor reliability of outputs (n=67, 28.15%) (Table 2).

**Table 2:** AI-related characteristics and utilization patterns among study participants (n=310) *Multiple responses; Questions on which artificial intelligence (AI) tools used, type of AI tool, frequency of AI tool use, previous AI training or exposure, source of information, and reported difficulties were answered only by participants who had used AI tools (n=238).

Variable	Frequency	Percentage
Ever used an AI tool for medical learning during the past 12 months	-	-
Yes	238	76.77%
No	72	23.23%
If yes, which AI tools were used? *	-	-
ChatGPT	221	92.86%
Microsoft Copilot	11	4.62%
Gemini	114	47.90%
Google Assistant	108	45.38%
Grok	17	7.14%
Perplexity AI	21	8.82%
Other	35	14.71%
Type of AI tool used	-	-
Free	227	95.38%
Subscription-based	11	4.62%
Frequency of AI tool use	-	-
Daily	86	36.13%
Alternate day	49	20.59%
Weekly	19	7.98%
Occasionally	84	35.29%
Previous AI training or exposure (training, workshop, orientation, or others)	-	-
Yes	63	26.47%
No	175	73.53%
Source of information about AI tools and their use in medical education*	-	-
Family	29	12.18%
Journal / textbook	32	13.45%
Newspaper	26	10.92%
Teaching or training in AI	54	22.69%
Television	36	15.13%
Social media	192	80.67%
Friends	131	55.04%
Other	36	15.13%
Reported difficulties while using AI tools *	-	-
Faulty data	72	30.25%
Paid versions needed	159	66.81%
Poor reliability of outputs	67	28.15%
Privacy concerns	79	33.19%
Unable to interpret results	44	18.49%
None	30	12.61%
Other	23	9.66%

Because the MAIRS-MS was designed for students with prior AI experience, readiness analyses were restricted to the 238 participants who reported using AI tools in the preceding 12 months; the perception analysis was conducted on the full sample of 310 students. The mean MAIRS-MS score was 71.75 ± 14.09 out of a maximum possible score of 110. Based on cut-off criteria, 169 of 238 participants (71.0%) were classified as having average AI readiness. The four MAIRS-MS subscales assess distinct dimensions of readiness: cognition (conceptual knowledge of AI), ability (confidence and competence in using AI tools), vision (understanding of AI's broader future role in healthcare), and ethics (awareness of ethical responsibilities and data privacy in AI use). Higher scores on each subscale indicate greater readiness in that respective domain. The mean subscale scores were 25.23 ± 5.57 for cognition, 26.66 ± 5.77 for ability, 9.82 ± 2.36 for vision, and 10.04 ± 2.28 for ethics. The mean perception score was 34.66 ± 7.07 (Table 3).

**Table 3:** MAIRS-MS and perception score among study participants (n=238) MAIRS-MS: Medical Artificial Intelligence Readiness Scale for Medical Students; SD: Standard deviation; MAIRS-MS scores were calculated among AI users (N=238), whereas the perception score was calculated among all participants (n=310).

Scale / Subscale	Mean ± SD
Cognition	25.23 ± 5.57
Ability	26.66 ± 5.77
Vision	9.82 ± 2.36
Ethics	10.04 ± 2.28
Total MAIRS-MS score	71.75 ± 14.09
Perception score	34.66 ± 7.07

Previous AI training showed a statistically significant association with the ethics domain of readiness (χ²=6.33, p=0.042), whereas other sociodemographic and AI-related variables did not demonstrate any statistically significant association with readiness (p>0.05). This suggests that students with prior structured exposure to AI demonstrated comparatively higher readiness in the ethics domain, which encompasses awareness of ethical responsibilities, data privacy, and accountability, indicating that ethical AI readiness may be particularly responsive to formal educational exposure (Table 4).

**Table 4:** Association of demographic and AI-related variables with AI readiness domains (n=238) ^*^Fisher’s exact test; AI readiness categories were based on cut-off criteria of the MAIRS-MS total score: poor (≤66), average (67–78) and good (≥79); AI: Artificial intelligence; χ²: Pearson’s chi-square test; p = p-value; p<0.05 was considered statistically significant.

Variable	Cognition χ² (p)	Ability χ² (p)	Vision χ² (p)	Ethics χ² (p)	Total readiness χ² (p)
Age	1.13 (0.568)	0.368 (0.832)	1.45 (0.930)	0.067 (0.967)	0.178 (0.915)
Gender	0.489 (0.783)	0.655 (0.721)	1.421 (0.491)	0.865 (0.649)	0.405 (0.817)
Place of residence	0.134 (0.935)	1.464 (0.481)	0.319 (0.852)	0.022 (0.989)	0.588 (0.745)
Academic year of study	1.847(0.933)	6.086 (0.414)	4.237 (0.645)	8.430 (0.208)	7.479 (0.279)
Type of AI tool used^*^	1.351 (0.496)	2.126 (0.351)	1.976 (0.337)	3.750 (0.116)	2.449 (0.242)
Frequency of AI tool use	8.826 (0.184)	8.245 (0.221)	7.498 (0.277)	10.065 (0.122)	6.446 (0.375)
Previous AI training	0.066 (0.968)	0.202 (0.904)	1.352 (0.509)	6.33 (0.042)	2.066 (0.356)

Spearman’s rank correlation showed an extremely weak positive correlation of total readiness score with age (r=0.087, p=0.181) and academic year of study (r=0.057, p=0.381); neither correlation was statistically significant (p>0.05) (Table 5).

**Table 5:** Correlation between age, academic year, and AI readiness domain and total scores (n=238) AI: Artificial intelligence; r: Spearman's rank correlation coefficient; p: p-value; p<0.05 was considered statistically significant.

Variable	Cognition r (p)	Ability r (p)	Vision r (p)	Ethics r (p)	Total Readiness r (p)
Age (years)	0.117 (0.072)	0.036 (0.576)	0.079 (0.224)	0.050 (0.442)	0.087 (0.181)
Academic year of study	0.114 (0.081)	−0.014 (0.827)	0.041 (0.531)	0.000 (0.998)	0.057 (0.381)

More than half of the students perceived that AI is useful for teaching (n=165, 53.23%), assignment preparation (n=162, 52.26%), self-learning (n=166, 53.55%), understanding complex concepts (n=170, 54.84%), and clinical case scenarios (n=161, 51.93%). However, many students also expressed concerns regarding misleading information (n=151, 48.70%), the potential impact of AI on clinical skills and critical thinking (n=151, 48.71%), and data privacy issues (n=133, 42.90%). Notably, a substantial proportion of participants selected the neutral response on nearly every item, ranging from n=115 (37.10%) to n=147 (47.42%), including n=137 (44.19%) who were neutral about AI improving curriculum quality. This pattern suggests that a considerable segment of students had not yet formed a clear opinion on several aspects of AI (Table 6).

**Table 6:** Perception of participants toward artificial intelligence (AI) (n=310) SA: strongly agree; A: agree; N: neutral; D: disagree; SD: strongly disagree; n (%): Frequency (Percentage).

Perception statements	SD	D	N	A	SA
-	n (%)	n (%)	n (%)	n (%)	n (%)
AI-powered platforms can improve online medical teaching and discussions	14 (4.52)	15 (4.84)	116 (37.42)	124 (40.00)	41 (13.23)
AI can provide quick and accurate feedback for assignments preparation & assessments	8 (2.58)	18 (5.81)	122 (39.35)	128 (41.29)	34 (10.97)
AI-based tools (like virtual simulations or chatbots) can enhance self-learning	11 (3.55)	11 (3.55)	122 (39.35)	135 (43.55)	31 (10.00)
AI is reliable for analyzing large amounts of medical data accurately without any error	17 (5.48)	41 (13.23)	144 (46.45)	86 (27.74)	22 (7.10)
AI can be useful in medical education for learning complex concepts and saving time	10 (3.23)	15 (4.84)	115 (37.10)	125 (40.32)	45 (14.52)
AI can help in preparing and explaining clinical case scenarios	12 (3.87)	15 (4.84)	122 (39.35)	136 (43.87)	25 (8.06)
Over-reliance on AI may negatively affect the development of clinical skills and critical thinking ability	9 (2.90)	15 (4.84)	135 (43.55)	93 (30.00)	58 (18.71)
AI may sometimes provide misleading or inaccurate information	11 (3.55)	19 (6.13)	129 (41.61)	122 (39.35)	29 (9.35)
AI poses challenges related to data privacy and confidentiality in medical education	11 (3.55)	19 (6.13)	147 (47.42)	102 (32.90)	31 (10.00)
Including AI in the medical curriculum will improve the quality of medical education	11 (3.55)	18 (5.81)	137 (44.19)	113 (36.45)	31 (10.00)

## Discussion

This study examined AI readiness and perceptions among undergraduate medical students at a tertiary care teaching institute in Gujarat. Overall, participants demonstrated an average level of AI readiness alongside generally positive perceptions of AI in medical education. However, gaps were identified in ethical understanding, formal AI training, and awareness of the limitations of AI, highlighting areas that may benefit from greater emphasis within undergraduate medical education.

The mean MAIRS-MS score of 71.75 ± 14.09 (out of 110) placed participants in the average readiness category, with approximately 71% of AI users demonstrating an average level of AI readiness. It is important to note that the MAIRS-MS was administered only to the 238 participants (76.77%) who reported prior use of AI tools, as the scale was developed for students with existing AI experience. The 72 students (23.23%) who had not used AI tools were excluded from readiness analysis. Since non-users are likely to have lower AI readiness than users, the mean MAIRS-MS score and the 'average readiness' conclusion represent the more experienced segment of the student population. The true overall readiness of the entire cohort may therefore be somewhat lower than reported, and this selection should be considered when interpreting these findings. These findings suggest that, although students have acquired a basic level of readiness to engage with AI technologies, there remains considerable scope to strengthen their competencies. Karaca et al. emphasized that AI readiness extends beyond familiarity with AI tools and encompasses the ability to critically evaluate, appropriately apply, and ethically use AI in medical practice [12]. Dhurandhar et al. reported a comparable pattern among medical students in central India, where moderate readiness coexisted with extensive self-reported AI use [17]. Studies from Indonesia and Malaysia have similarly described a combination of positive attitudes and measurable gaps in practical knowledge and structured training [14,15]. The convergence of these findings across geographically and institutionally diverse settings implies a systemic mismatch: students are integrating AI into their academic lives at a pace that medical school curricula have yet to match. Enthusiasm and tool adoption, while encouraging, do not by themselves produce the competency framework required for responsible clinical AI use.

Subscale analysis demonstrated variation in AI readiness across the four MAIRS-MS domains. Participants achieved higher mean scores in the cognition (25.23 ± 5.57) and ability (26.66 ± 5.77) domains, suggesting greater familiarity with AI concepts and greater confidence in using AI-assisted tools during learning activities. In contrast, lower scores in the vision (9.82 ± 2.36) and ethics (10.04 ± 2.28) domains indicate comparatively limited preparedness regarding the broader implications of AI, including its future role in healthcare, ethical considerations, responsible use, and data privacy.

The observed pattern of higher scores in the cognition and ability domains than in the vision and ethics domains has also been reported in several previous studies. Karaca et al. and Dhurandhar et al. similarly found that knowledge- and skill-oriented domains scored higher than the ethics domain among medical students [12,17]. Comparable findings were reported by AlZaabi and Masters among pre-clinical medical students in the United Arab Emirates, where higher proficiency in technical AI-related domains contrasted with lower performance in the ethics domain [16]. Similarly, Yazdi et al. reported lower levels of ethical awareness than practical AI competence among dental faculty members and students [23]. These converging findings underscore that any curriculum model limited to technical AI skills will be insufficient; formal instruction should also include ethical principles, AI governance, accountability, and patient data privacy.

The high prevalence of AI tool use (76.77%) in this cohort, with ChatGPT being the most commonly used platform (92.86%), indicates that generative AI has become widely integrated into students' learning practices. However, most participants relied on freely available AI tools (95.38%) and social media (80.67%) as their primary sources of AI-related information, whereas only 26.47% had received formal AI training.

Similar patterns have been reported by Ahmad et al. among medical students in Jharkhand, India, and by Lugito et al. in Indonesia, where students primarily relied on freely available AI tools and self-directed learning rather than structured educational programs [10,14]. The coexistence of widespread AI use and limited formal training (26.47%) suggests that many students may be acquiring AI-related knowledge through informal sources, potentially reinforcing misconceptions about AI capabilities and limitations.

Participants also identified several practical barriers to effective AI use. The most frequently cited difficulty was the need for paid subscriptions to access advanced features (66.81%), followed by concerns about faulty or inaccurate data (30.25%) and poor reliability of AI-generated outputs (28.15%). Privacy concerns were raised by roughly one-third of users. McCoy et al. similarly documented that both students and faculty recognized deficiencies in AI education and advocated for structured, curriculum-based training to address practical challenges in AI use [24]. Collectively, these observations suggest a disconnect: although generative AI has achieved rapid uptake among students, the limited availability of structured institutional AI education, as reflected by only 26.47% of participants having received any formal AI training in this study, means that knowledge acquisition remains largely opportunistic and unsystematic, which may not adequately support the development of rigorous, evidence-informed clinical reasoning.

Students generally viewed AI as a useful tool in medical education. Most participants considered AI beneficial for teaching, assignment preparation, self-learning, understanding complex concepts, and explaining clinical case scenarios. At the same time, many students expressed concerns about misleading information, excessive reliance on AI, its effect on clinical skills and critical thinking, and data privacy.

Although direct comparison of the mean perception score with previous studies was not appropriate because different questionnaires were used, the overall pattern of responses was consistent with earlier reports. Huisman et al. found that radiologists and radiology residents internationally held similarly dual attitudes, appreciating AI's diagnostic contributions while remaining anxious about professional displacement [6]. In the Indian healthcare context, Tezpal et al. documented receptivity toward AI among practitioners tempered by concerns about output reliability and the hazards of over-dependence [21]. Sarkar et al. observed comparable ambivalence among undergraduate students, with enthusiasm for AI coexisting with concern about misinformation and erosion of clinical judgment [18]. That our student cohort mirrors this pattern despite limited formal training suggests that students are developing nuanced, experience-informed views of AI primarily through self-directed use, underscoring the need for structured frameworks that help them evaluate AI outputs rather than simply consume them.

Chi-square analysis across all variables revealed a single statistically significant relationship: prior AI training was associated with higher readiness specifically in the ethics domain (χ²=6.33, p=0.042). All other variables tested (gender, place of residence, academic year, type of AI tool preferred, and frequency of AI use) showed no significant association with readiness in any domain or in the total score (all p>0.05). Spearman's correlation analysis similarly yielded no significant relationship between age or academic year and readiness domain scores.

The statistically significant association between prior AI training and higher ethics domain readiness raises the hypothesis that structured educational exposure to ethical dimensions of AI may enhance readiness in this domain, though the cross-sectional design precludes any causal inference, and longitudinal studies are needed to confirm this relationship. The inclusion of ethics as a core domain in the MAIRS-MS developed by Karaca et al. underscores its importance as a key component of AI readiness [12]. Nonetheless, the present findings are consistent with the potential value of integrating structured AI education into undergraduate medical curricula. AlZaabi and Masters corroborated this by demonstrating that structured training produced more consistent gains in ethical reasoning than did general AI familiarity alone [16]. This observation is not limited to medical students. Studies among educators and faculty have reported similar findings: Alnasib found that faculty members in Saudi Arabia who had received prior AI training demonstrated greater willingness to incorporate AI into their teaching [25], while Bukhari et al. reported that training exposure was a key factor differentiating levels of institutional readiness among medical college faculty in Pakistan [26]. Comparable patterns have been noted in dental education, where faculty preparedness varied considerably based on prior formal training [27], and in broader higher education contexts, where faculty attitudes toward AI adoption were closely linked to the availability of structured training opportunities [28]. These consistent findings across different professional groups and educational levels reinforce the case that even brief, formally structured AI training meaningfully improves readiness, especially in its ethical dimension.

Conversely, the absence of significant associations between readiness and sociodemographic variables indicates that demographic factors alone do not predict how prepared a student is to engage with AI. This is consistent with studies by Dhurandhar et al. and Tung and Dong, both of which found that readiness was shaped more by educational exposure and learning experiences than by background characteristics [15,17].

Notably, frequency of AI tool use was not correlated with readiness scores in any domain, suggesting that volume of use and depth of understanding operate as distinct dimensions. Students who use AI tools frequently, primarily for task completion such as generating assignment content or summarizing lecture material, may do so without engaging with the conceptual or evaluative aspects of the technology. This form of instrumental use does not require, nor does it necessarily develop, an understanding of how AI systems function, where their outputs may be unreliable, or what ethical responsibilities accompany their use in clinical contexts. Depth of understanding appears to require deliberate, structured instruction rather than repeated informal exposure alone.

Strengths and limitations

This study assessed AI readiness and perceptions among undergraduate medical students at a tertiary care teaching institute in Gujarat, where published evidence on this topic remains limited. AI readiness was evaluated using the MAIRS-MS, a psychometrically validated instrument. Perceptions toward AI were assessed using a faculty-developed questionnaire that demonstrated excellent internal consistency (Cronbach's α =0.933), providing complementary information on students' attitudes toward AI. Furthermore, the inclusion of students from all MBBS years and the use of a census sampling approach, together with a 77.5% response rate, enhanced the representativeness of the study population and strengthened the robustness of the findings.

Several limitations should be considered when interpreting the findings of this study. First, the cross-sectional design provides a snapshot of AI readiness and perceptions at a single point in time and does not permit the establishment of causal relationships between the variables studied. Second, the use of a self-administered online questionnaire may have introduced response bias, as participants' self-reported readiness and perceptions may not fully reflect their actual knowledge or competencies. Third, because the study was conducted at a single tertiary care teaching institute in Gujarat, the findings may not be generalizable to medical colleges with different student populations, educational environments, or institutional resources. The use of percentile-based, sample-dependent cut-offs (33.33rd and 66.67th percentiles) to classify readiness as poor, average, or good is a limitation, as these thresholds are derived from the observed distribution of the present sample and may not be directly comparable to classifications used in other studies employing different cut-off approaches or future studies using standardized normative values. Finally, AI readiness was assessed using self-reported responses rather than objective performance-based measures; future studies incorporating practical assessments or standardized competency-based evaluations would provide a more comprehensive understanding of students' preparedness to use AI in clinical practice.

## Conclusions

This study found that undergraduate medical students at a tertiary care teaching institute in Gujarat demonstrated an overall average level of AI readiness together with generally mixed-to-positive perceptions toward artificial intelligence, with a considerable proportion of students remaining neutral across several items. Among the variables examined, previous AI training was the only factor significantly associated with AI readiness, specifically within the ethics domain, whereas only an extremely weak, non-significant positive correlation was observed between AI readiness and the selected sociodemographic variables. More than half of the students perceived AI as a useful educational tool for teaching, assignment preparation, self-learning, understanding complex concepts, and clinical case scenarios. However, many also expressed concerns regarding misleading information, the potential impact of AI on clinical skills and critical thinking, and data privacy. The findings of this study suggest that incorporating structured AI education, with particular emphasis on ethical principles, may strengthen undergraduate medical students' readiness for the appropriate use of AI in healthcare.
